# A sequence embedding method for enzyme optimal condition analysis

**DOI:** 10.1186/s12859-020-03851-5

**Published:** 2020-11-10

**Authors:** Xiangjun Li, Zhixin Dou, Yuqing Sun, Lushan Wang, Bin Gong, Lin Wan

**Affiliations:** 1 School of Software, Shandong University, Shunhua Road, Jinan, 250101 China; 2 State Key Laboratory of Microbial Technology, Shandong University, Binhai Road, Qingdao, 266237 China

**Keywords:** Protein sequence analysis, Embedding, Bioinformatics

## Abstract

**Background:**

An enzyme activity is influenced by the external environment. It is important to have an enzyme remain high activity in a specific condition. A usual way is to first determine the optimal condition of an enzyme by either the gradient test or by tertiary structure, and then to use protein engineering to mutate a wild type enzyme for a higher activity in an expected condition.

**Results:**

In this paper, we investigate the optimal condition of an enzyme by directly analyzing the sequence. We propose an embedding method to represent the amino acids and the structural information as vectors in the latent space. These vectors contain information about the correlations between amino acids and sites in the aligned amino acid sequences, as well as the correlation with the optimal condition. We crawled and processed the amino acid sequences in the glycoside hydrolase GH11 family, and got 125 amino acid sequences with optimal pH condition. We used probabilistic approximation method to implement the embedding learning method on these samples. Based on these embedding vectors, we design a computational score to determine which one has a better optimal condition for two given amino acid sequences and achieves the accuracy 80% on the test proteins in the same family. We also give the mutation suggestion such that it has a higher activity in an expected environment, which is consistent with the previously professional wet experiments and analysis.

**Conclusion:**

A new computational method is proposed for the sequence based on the enzyme optimal condition analysis. Compared with the traditional process that involves a lot of wet experiments and requires multiple mutations, this method can give recommendations on the direction and location of amino acid substitution with reference significance for an expected condition in an efficient and effective way.

## Background

Proteins are made up of hundreds of monomers called amino acids that are attached to one another by peptide bonds, forming a long chain defined as primary structure. It further constructs the second structure and the tertiary structure of the protein, which finally determines the protein function and the optimal condition of its activity [1], such as the alkali resistance, or the salt tolerance.

Enzymes are a kind of catalytic proteins. The enzymes with the same function are referred as a family, which have some identical or conserved amino acid fragments in sequences that are not prone to mutate. Although these enzymes have the same biological function, some of them are more active than others at the same alkaline or temperature condition, which are caused by the different parts in the sequences, defined as the non-conserved fragments. For example, the enzymes in GH11 family can degradate the heteroxylans that constitute the plant cell wall in lignocellulosic biomass, but they have different optimal temperatures or pHs [2]. In many practical applications, we need to find the most active enzyme in a family under a given condition or to select an enzyme for a higher activity than the existing enzymes by directed evolution. For these purposes, biological researchers generally perform a series of gradient tests to measure the optimal condition of each enzyme in a family. Then they adopt the protein engineering to produce an enzyme with an expected optimal condition [3]. But the above biological methods analyze only one enzyme at each time such that they cost much time, power and resources to get the expected enzyme.

Recently, many works have employed machine learning methods to analyze the optimal temperature or pH for enzymes [4]. For example, Dijk et al. used the ratio between the number of residues inside and outside the folding structure of protein as the measure for its hydrophobicity so as to deduce the temperature dependence of the protein [5]. Pucci et al. used temperature-dependent statistical potentials to compute the fold free energies at three different temperatures, and used Gibbs–Helmholtz equation to predict the protein thermal stability curve [6]. The SCooP method predicted the full T-dependent stability curve by protein structures and host organisms [7]. However, these methods require the tertiary structure [8], free energy [9], etc., which are not easy to obtain in practice. Another representative method is based on the correlations between the tertiary structure and free energy^1^ of an enzyme. It judges the stability of the mutant by analyzing the change of free energy in this process [10]. For example, Dehouck et al. used a linear combination of statistical potentials whose coefficients depend on the solvent accessibility of the mutated residues to predict the stability changes caused by single site mutations in proteins [11]. Wijma et al. created a library of potentially stable mutations by calculating the change in the free energy of mutations, and reduced the size of the library by eliminating false positive predictions and chose the most stable combination of mutations to mutate [12]. These methods are based on molecular dynamics to simulate the state of proteins such that they perform a single protein in one experiment. But it can not analyze a group of proteins at the same time.

In this paper, we propose an embedding method to determine which one has a better optimal condition for two given sequences directly using the amino acid sequences. Amino acids and the construct information in sequence are represented as vectors in the same latent space, which are learned by the compatibility between the sites and expected condition. We propose the compatibility objectives on both a single-site with an amino acid and on multiple sites with different amino acids. Based on these vectors, we can predict whether a new enzyme has a better optimal condition than a wild enzyme by analyzing its sequence information. We select the enzymes in the GH11 family as the practical usage whose optimal pH values are already determined. Since from the view of machine learning the quantity of samples is small, we use the statistics to approximate the probability distribution on amino acids on each site of the aligned sequences and generate more samples for the embedding training.

Based on these embeddings, we analyzed the non-conserved segment and the conserved segment of these enzymes. These results are consistent with the results on WebLogo^2^ [13, 14]. We then design two experiments for biological purposes. One is to compare two enzymes which has a higher optimal pH or which has a higher activity in the same pH environment. Another is to quantify the probability that after introducing mutations into a given enzyme sequence, whether the enzyme still have high activity for an expected optimal condition. Our experimental results are consistent with the biological results. Comparing with other methods, the embedding method is more efficient and effective.

## Notions and dataset

### Notions

There are 20 kinds of amino acids commonly used forming proteins in nature. Let *aa* denotes the set of amino acids in bioinformatics, i.e. $aa=\{Ala, Arg, Asp, Cys,$ $Gln, Glu, His, Ile, Gly, Asn, Leu, Lys, Met, Phe, Pro, Ser, Thr, Trp, Tyr, Val\}$. Since the amino acid sequences in a family are often with different lengths, they are aligned before analysis. After alignment, the identical or similar segments are in the successive columns, which are helpful to find the functional or evolutionary structure. We denote the gap between amino acids as a symbol $'-'$ and the set of elements consisting aligned amino acid sequences are replaced by $A=\{-\}\cup aa$. Given a family of amino acid sequences, denoted by ${\mathbf {F}}$, the length after alignment is denoted by *l*. For an amino acid sequence, we use *k* to indicate the $k{\rm th}$ site of the sequence, $c_k$ to represent the amino acid at the $k{\rm th}$ site, and $c_k^a$ for the amino acid of the specific amino acid sequence *a* at site *k*.

To learn the correlations between a sequence and the optimal condition, we introduce two metrics. The first is the correlation between a single-site with an amino acid in an aligned sequence. We define the event (*k*, *a*) as amino acid $c_k^a\in A$ being on the $k{\rm th}, k\in [1..l]$ site of an aligned sequence ${a}\in {\mathbf {F}}$, and introduce the metric of *capability score* *s*(*k*, *a*) to evaluate how this event influences the optimal condition. Taking alkali resistance as an example, if the *capability score* is higher when amino acid $c_k$ appears at site *k*, it indicates that the occurrence of this event leads to an increase in the optimal pH of the sequence.

Another is the correlation between the optimal condition and multiple amino acids appearing on different sites so as to measure how multiple elements together affect the optimal condition. We define the event $(n_i^a,n_j^a)$ as $c_i^a$ appears on site *i* and $c_j^a$ appears on site *j* in the sequence $a\in {\mathbf {F}}$. Then we introduce the *suitability score* $s(n_i^a,n_j^a)$ to denote how much this event induces a better optimal condition. Different with the single site analysis, the site *k* and the amino acid $c_k^a$ on it are considered together by concatenating their vectors.

A higher score means that the occurrence of event $(n_i^a,n_j^a)$ induces a better optimal condition.

To combine these two objectives together, a hyper parameter $\alpha \in (0,1)$ is introduced to balance them in the embedding learning process. Let ${\mathcal {L}}_T(\theta )$ and ${\mathcal {L}}_C(\theta )$ denote the objective functions for the above scores in the embedding learning process, respectively, where $\theta$ denotes all the parameters to be learnd. The whole objective function is then defined as ${\mathcal {L}}(\theta )=\alpha \cdot {\mathcal {L}}_T(\theta )+(1-\alpha )\cdot {\mathcal {L}}_C(\theta )$. A larger $\alpha$ induces a higher bias on *s*(*k*, *a*). Conversely, a smaller $\alpha$ considers more on the correlations between the optimal condition and multiple amino acids appearing on different sites. We would discuss how to learn the embeddings in the "Method" section.

### Dataset

We crawl the amino acid sequences of the GH11 family on the CAZy website^3^. In order to extract the required optimal pH of each sequence, we investigate the papers related to the proteins in the GH11 family. There are 125 amino acid sequences with optimal pH condition in the GH11 family and the length of each sequence is within 128 and 335. After alignment, the length of amino acid sequences is $l=380$. We choose the higher alkali-resistance as the expected condition.

To learn the embeddings of amino acid sequences, we partition ${\mathbf {F}}$ into two subsets. Let $S_l\subset {\mathbf {F}}$ denote the collection of sequences whose optimal pH values are lower than the average and $S_h\subset {\mathbf {F}}$ denote the collection of sequences whose optimal pH values are higher than the average, respectively.

**Fig. 1:**
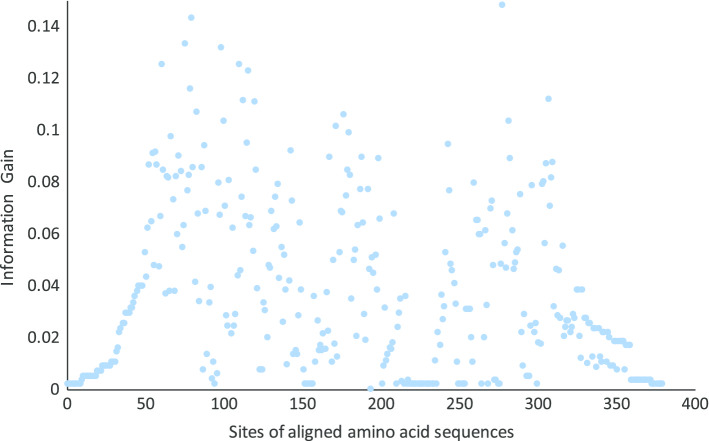
Information gain for the sites in aligned amino acid sequences in GH11 family

To select the appropriate sites for rational design, we take into account the conserved and non-conserved segments in the aligned sequences, and quantify the importance of each site against the optimal pH, namely which sites the amino acids being on highly influence the alkali resistance. We adopt the information gain as a metric. Give a sequence *x*, the information entropy *H*(*y*) quantify the uncertainty on whether $x's$ optimal pH *y* is either higher or lower than the average, i.e. $H(y)=-\sum _{i\in \{l,h\}}{p(x\in S_{i})\cdot \log p(x\in S_{i})}$, where $p(x\in S_{i})=\frac{|S_i|}{|{\mathbf {F}}|}$. Then we quantify how much a concrete site reduces this uncertainty. For site *k*, the conditional entropy $H(y|c_k)$ quantities the uncertainty on the optimal pH after we know the amino acid $c_k$, i.e $H(y|c_k)=-\sum _{i\in \{l,h\}}{p(x\in S_{i}|c_k)\cdot \log p(x\in S_{i}|c_k)}$. The conditional entropy on site *k* is the expectation on different amino acids, i.e. $H(y|k)=\sum _{c_k\in A}\frac{|F_{c_k}|}{|{\mathbf {F}}|}\cdot H(y|c_k)$, where $F_{c_k}$ denotes the set of sequences with the amino acid $c_k$ on site *k*. The information gain on site *k* is $Gain(k)=H(y)-H(y|k)$. The higher this score, the more probability different amino acids on site *k* influences the optimal condition. The results on GH11 family are scatter plotted in Fig. 1, where the X-axis denotes sites of aligned amino acid sequences and Y-axis denotes the information gain on a site. This point would be considered together with a concrete amino acid for mutation, discussed in the next section.

## Results

In this section, we discuss how to use the learnt embeddings for biological purposes so as to help biologists for efficient analysis without wet experiments. We consider the important task on an enzyme mutant for an expected optimal condition, which is better than others in a family. A usual way is first to verify the optimal condition of each enzyme in a family by wet experiments and select the one that is most approaching the expected condition. Then biologists introduced some mutations in the non-conserved regions and conducted wet experiments to verify the activity of the mutant under the expected environment, known as the rational design. These biological purposes can be transferred as the following two computational problems: the pH predication and mutation recommendations.

### Sequence based optimal pH predication

Given two amino acid sequences in a family, we aim to determine by computation which one has a better optimal condition, such as a higher alkali resistance. We first verify whether a general regression is appropriate for the pH prediction task using the learned embeddings. For this purpose, we calculate the *capability score* on each site for a given sequence $a\in {\mathbf {F}}$ by the learnt embeddings as the input of our model. Then we predict the value of optimal pH $\hat{y}_a$ and calculate the residual by $\hat{y}_a-y_a$ with its real optimal pH $y_a$. We try different prediction models, and finally choose the Support Vector Regression (SVR) [15] method as the prediction model, where the kernel function is *rbf*, punishment term is 10, and the parameter gamma of *rbf* is 0.0001. The input of the SVR model is the feature vector of the amino acid sequence and the output is the optimal pH.

Since the current related works of predicting the optimal condition are based on the tertiary structure and/or free energy, there is not any closely related works on the sequence based predication. Therefore, we select several machine leaning algorithms as the comparison methods.

- SVR [15]. This is the traditional predication method. We try several kernel functions, such as *rbf*, *poly*, *linear* and *sigmoid*, respectively, and adjust the parameters to verify the effects of the SVR. We select the best results from several variants. The corresponding parameters include: *poly* as the kernel function, 10 as the punishment term C, 3 as the dimensions of poly functions.
- Neural network (NN) [16]. There are many factors affect the results of the Neural Network method, such as the activation function, optimization function, the number of hidden layer and the number of nodes on the neural network model.We explore the effects on different settings and select the best one as: the number of nodes in hidden layer to 1000 in one hidden layer, *tanh* as the activation function and *adam* as the optimization function.

As a comparison to embeddings, we use the one-hot vector on the set *aa* of amino acids as the sample features to feed these methods. Namely, each site *k* of a sequence maps to a vector $x\in \{0,1\}^{l}$ and $x_i=1$ indicates the amino acid $c_k$ is at rank *i* in the lexicographical order of the set of amino acids. The gap maps to zero in a vector.

**Fig. 2:**
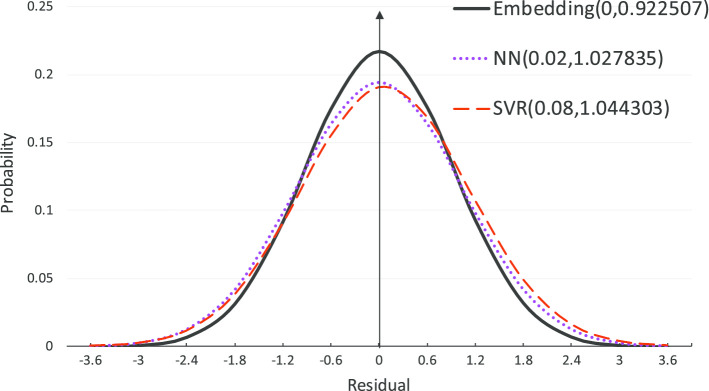
Probability distribution on the predication residuals $\hat{y} - y$. The $\hat{y}$ is the predicted value of optimal pH for a given sequence, and the *y* is the real optimal pH

As the results shown in Fig. 2, we can see that, on one side, the residuals by the embedding model follow the Gaussian distribution G(0, 0.9225), which is better than the comparison methods. On the other side, although the best result has a very small variance 0.9225, it is still not good enough by taking into account the biology context of an enzyme mutant. Since a mutant is expected to survive in higher pH condition such as 1 pH larger than before, the statistics only convinces about 35% cases satisfying this condition, i.e. $\mu + \sigma$. The reason that results in this unaccepted result is that from the perspective of machine learning the amount of labelled data, i.e the 125 amino acid sequences with labels of optimal pH condition in the GH11 family, are far less than required, which makes the model over-fitting and less robust.

**Fig. 3:**
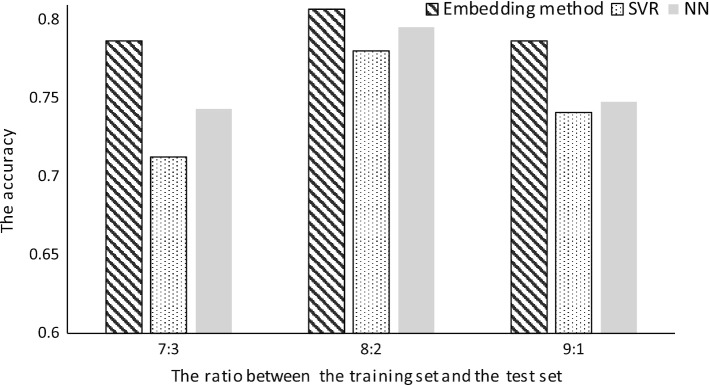
The accuracy of the embedding method and comparison methods in different ratios on training data

Thus it is not appropriate to directly predict the pH value for a given sequence although the introduce of embedding to amino acid sequence and site help the optimal condition analysis. Then we adopt the ranking metric for pH predication. For two given amino acid sequences, we predict which one has a better optimal condition by comparing their pH values. We use the accuracy as the evaluation criteria. If the result is consistent with the real ranking between them, it is a correct judgment. Otherwise, it is incorrect. The accuracy is computed by the ratio of correct judgments against the number of independent experiments. By a series of this ranking comparison, we can quickly find an appropriate one from a candidate enzyme set.

In this experiment, we also compare with other methods and show the results in Fig. 3, together with several settings on the ratio between the training set and the test set. The threshold of the information gain $\omega$ is set 0.0478, and the dimension of embeddings is set 30 and the preference of the combinative objectives $\alpha$ is set 0.4. We use the 10-fold cross-validation method to verify the accuracy, and adopt the average of 10 repeated experiments for each ratio. The results show that the embedding method gives the best results in most cases comparing with other methods.

Consequently, in consideration of the current size of the data set and the above results, we choose to use a comparison method to give reasonable and constructive opinions on the rational design of enzymes.

### Experiment on the influence of parameters

In this subsection, we discuss the influence by the super parameter, including the dimensions of embeddings *d*, the preference of the combinative objectives $\alpha$ for embedding learning and the threshold of the information gain $\omega$ for selecting the sites on computation of score.

First, we discuss the influence of different dimensions of embeddings on the accuracy. We try several settings on the dimension of embeddings, i.e. $\hbox {d}=5, 10, 30, 50, 90$ and 130, respectively, and list the results in Table 1.

**Table 1:** Summary on the accuracy against different ratios on training data, the dimensions of embedding, and comparison methods

Train:test	Dimension of embedding vector					
	5	10	30	50	90	130
5:5	0.734	0.753	0.706	0.704	0.694	0.685
6:4	0.755	0.752	0.744	0.751	0.749	0.746
7:3	0.743	0.77	0.787	0.792	0.794	0.794
8:2	0.751	0.793	*0.813*	0.803	0.804	0.801
9:1	0.745	0.773	0.787	0.794	0.799	0.799

The best is denoted in italics

Considering the influence by the dimension of embeddings, we verify the accuracy against different settings. We can see that after the dimension is larger than a threshold 30, there is not obvious difference on accuracy. This illustrates that a small dimension cannot hold much implicit information of biological semantics on 20 amino acids and the sites of the aligned sequences. However, a too large dimension may induce the sparse problem and include too much noise. Consequently, it is difficult for convergence due to a small quantity of samples.

Considering the ratio of the training set and the test set, a larger ratio often induces a higher accuracy. Because when the ratio is small, the training set can not reflect the probability distribution of the whole dataset. But a dominate ratio, i.e. 90%, may induce the number of test set too small such that the results are with more randomness and can not reflect the representative results.

Since these experiments are performed only on real enzymes, the number of samples is small either on training set or testing set, i.e. totally 125 amino acid sequences with optimal pH condition in the GH11 family. However, the enzyme activity and optimal conditions are different from each other. The contingency in each experiment is unavoidable. So, we further analyze the stability of performances under different settings by the standard deviation of the results. In most cases, the standard deviation of performances under each setting is within [0.005, 0.052]. Under the setting of ratio 8:2 and dimension 50, the performances are most stable with a low standard deviation 0.007 and a high accuracy 0.803. Under the setting of ratio 8:2 and dimension 30, the accuracy expectation is the highest 0.813 with a low standard deviation 0.016. These settings are recommended.

**Fig. 4:**
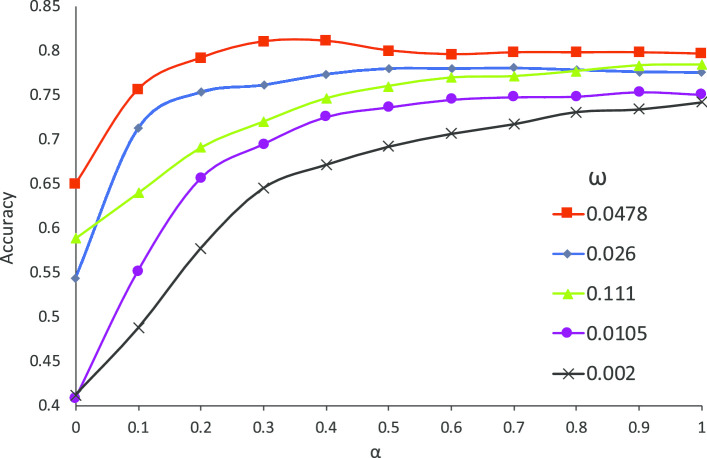
The accuracy against different parameter settings. Each curve represents the accuracy under the given threshold $\omega$

In the following experiments, the embedding dimension is set 30, and the ratio between training set to test set is 8:2. We choose the threshold $\omega$ as 0.111, 0.0478, 0.026, 0.0105 and 0.002, and $\alpha$ as 0, 0.1, 0.2, 0.3,..., 1, respectively. The accuracy results against different parameters, i.e. the balance factor $\alpha$ and the threshold $\omega$ on information gain, are shown in Fig. 4.

**Fig. 5:**
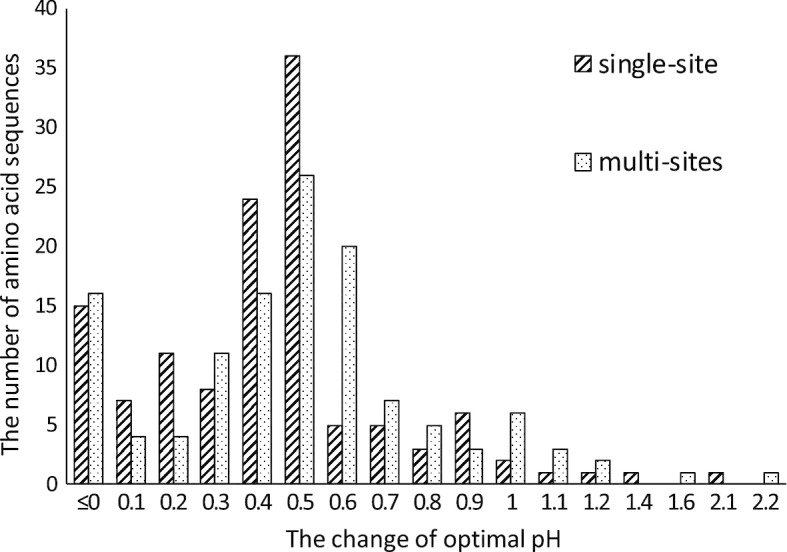
Statistical of the change of optimal pH. Each cylinder represents that how many mutants change their optimal pH. X-axis represents the change in optimal pH

Considering the threshold of the information gain $\omega$ for selecting the sites on computing the score, a suitable $\omega$ is required. The best setting is at $\omega =0.0478$, colored red. A large threshold on the information gain $\omega$ induces fewer sites being selected and some important non-conserved sites being ignored. Conversely a small $\omega$ may introduce more conserved sites in calculating the score such that it reduces the accuracy.

Considering the preference on $\alpha$, both the type of amino acids on single site and interactions between multiple sites contribute to accuracy. When $\alpha$ is at 0.4, i.e. the type of amino acids on single site, our method achieves the best. When $\alpha$ is less than 0.4, our optimization objectives would consider more interaction of different sites. The special case $\alpha =0$ learnt less about the semantics on amino acids, which only considers the correlations between the optimal condition and multiple amino acids appearing on different sites. However a larger $\alpha >0.4$ does not influence much on predication. Empirically, the appropriate setting is $\alpha =0.4$.

### Embedding based mutation suggestion

In this subsection, we discuss how to guide mutations on enzymes so as to help biologists wet experiments. Given a family of enzymes and an expected optimal condition like alkali-resistance, we design a new amino acid sequence such that it has a better optimal condition than the wild-type enzymes.

For a given wild-type enzyme, we propose two rules for a single-site mutation: (1) Do not mutate the site that is filled by gap $'-'$ so as to remain the original length of the amino acid sequence. (2) Do not mutate the sites with information gain smaller than 0.0478. Then we calculate the scores of different single-site mutations and select the mutant with the highest score. For multiple-sites mutation, we combine single-site mutations to get multi-sites mutants on a wild-type enzyme, and retain the mutant with the highest score.

We perform single-site and multi-sites mutations on the known wild-type amino acid sequences in GH11 family. From a biological point of view, the more mutation sites, the larger the uncertainty of the mutation and the higher the cost of mutation. So we only do at most 3-sites mutation for an amino acid sequence. We use Neural Network model to predict the optimal pH of mutants, and calculate the change of optimal pH after mutation. The results are shown in Fig. 5, where the X-axis represents the change of optimal pH and the Y-axis represents the number of amino acid sequence under the change.

For both mutations, the optimal pH of the 88% amino acid sequence increases. From the prediction results, both mutation suggestions can increase the optimal pH of amino acid sequences and the effect of multi-sites mutation suggestion is better than that of single-site mutation suggestion. Specially, The optimal pH of the 5% amino acid sequence in single-site mutation and 10% amino acid sequence in multi-sites mutation increase more than 1.0.

### Bioinformatics verification

In this subsection, we verify the computational results with the biological wet experiments. We take the enzyme xylanase A from the GH11 family as an example whose optimal pH is 6.0. Ruller et al. generated 5 mutants on xylanase A which can survive in alkaline environment by multiple mutation experiments and determine their activity at pH 8.0 environment by wet experiments [17]. We use Eq. 1 to calculate the activity scores of xylanase A and the 5 mutants, which consists of two parts: the compatibility of the amino acid on each site $s(k,a_k)$ and the influence induced by multiple amino acids on different sites $s(n_i^a,n_j^a)$. The higher the score, the higher the activity of the enzyme at the same pH environment.

1 $$\begin{aligned} Score(a)=\alpha \cdot \sum _{k=1}^ls(k,a_k)+(1-\alpha )\cdot \frac{\sum _{i,j=1}^ls(n_i^a,n_j^a)}{l} \end{aligned}$$

Both the activity at 8.0 pH [17] and the scores of these enzymes calculated by our method are together listed in Table 2. By comparing the changes in activity and scores between mutants and wild-type xylanase A, we can see that the trend of activity are consistent with the scores. These are positive correlations.

**Table 2:** The activity at pH 8.0 and the activity score on xylanase A and Mutant

Wild and Mutants	Mutation sites	Activity at pH 8.0	Score
Wild xylanase A	N/A	1.29 U/ml	4022.35
Mutant1	S22P	2.87 U/ml $\uparrow$	4024.73 $\uparrow$
Mutant2	G13R	0.99 U/ml $\downarrow$	4018.68 $\downarrow$
Mutant3	Q7H/G13R/S22P	1.71 U/ml $\uparrow$	4022.88 $\uparrow$
Mutant4	S22P/H56L/S179C	0.88 U/ml $\downarrow$	4024.73 $\uparrow$
Mutant5	S31Y	1.68 U/ml $\uparrow$	4022.35*

A special case is mutant5, which may be caused by the mutation on site 31. Since the information gain on this site is only 0.039 and is lower than the threshold 0.0478, it is not included in calculating the score. But considering the little change in activity after mutation, it is not important to select this mutant for an expected optimal condition.

## Discussion

### Understanding the biological semantics of embeddings

In this proposed method, the learnt embeddings are expected to include the biological semantics for an expected optimal condition. Since the embedding vectors are in the latent space, we adopt the distributed stochastic neighbor embedding (*t-SNE*) method [18] to reduce the high-dimension vectors to 2d coordinates for illustration.

**Fig. 6:**
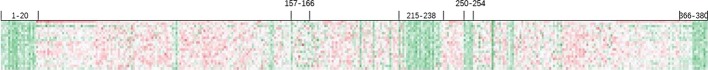
The thermodynamic chart for the capability scores on PH for the GH11 family. The X-axis represents the aligned 380 sites of sequences and the Y-axis represents amino acids and the gap in set A

We first verify whether the capability of amino acids with the sites of a sequence is embedded in the vectors. The learned *capability scores* *s*(*k*, *a*) are illustrated as the thermodynamic chart in Fig. 6, where the X-axis denotes the sites and Y-axis denote the amino acids. Red color maps to high scores, while green color maps to low scores. The green areas 1–20, 157–166, 215–238, 250–254 and 366–380 indicate amino acids on these sites influence less the optimal pH, while the red areas indicate that different amino acids on these sites highly influence the optimal pH. Compared with the results by biologist analysis, these areas are mapped to the conserved segments and the potential non-conserved segments, which is the basic task for analyzing the enzymes in a family. This convinces the semantics of embeddings.

**Fig. 7:**
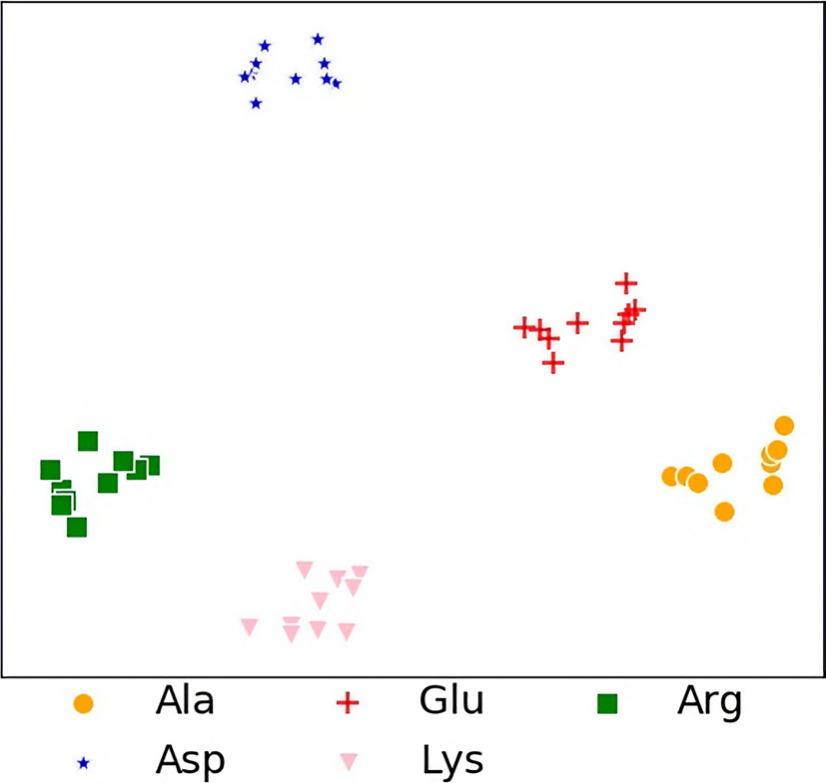
The $t-SNE$ coordinates of the embeddings. Each point denotes a combinative embedding by a site and an amino acid. The selected amino acids *Ala*, *Glu*, *Arg*, *Asp* and *Lys* are colored by orange, red, green, blue and pink, respectively

Then we verify the biological differences between amino acids. We select Top10 information gain sites and two alkali-resistance amino acids *Lys* and *Arg*, as well as two acid-resistances amino acids *Asp* and *Glu*. We combine the embeddings of each pair of them and color them as points in Fig. 7 by $t-SNE$. The results show that the points that are combined with the same amino acid are clustered together rather than others. Moreover, the points combined with the alkali-resistance amino acids, colored green and pink, are clustered more closely than with the acid-resistance amino acid, colored blue and red.

**Fig. 8:**
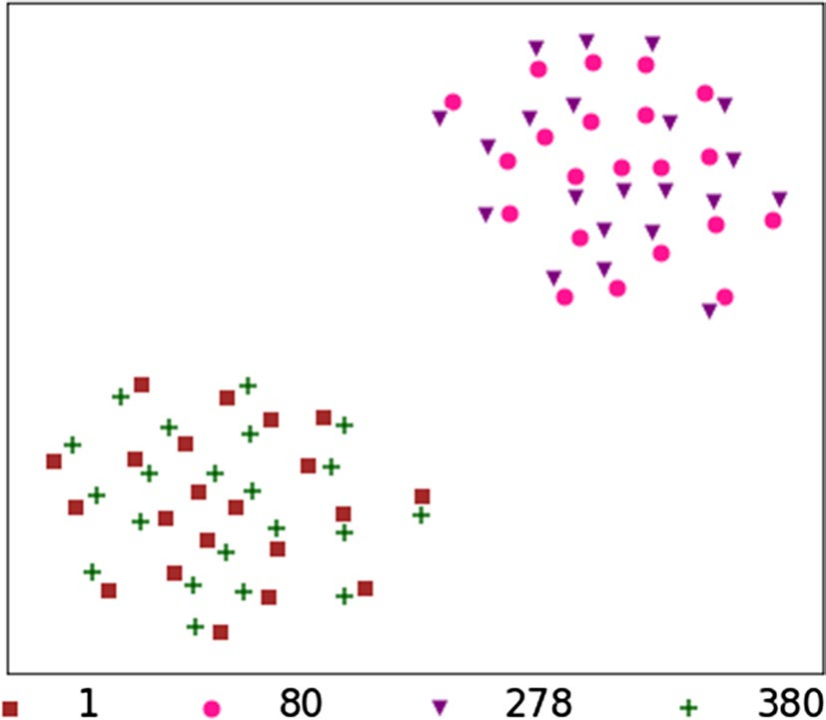
The $t-SNE$ coordinates of the embeddings. The sites 1, 80, 278 and 380 are colored brown, pink, purple and green, respectively

**Fig. 9:**
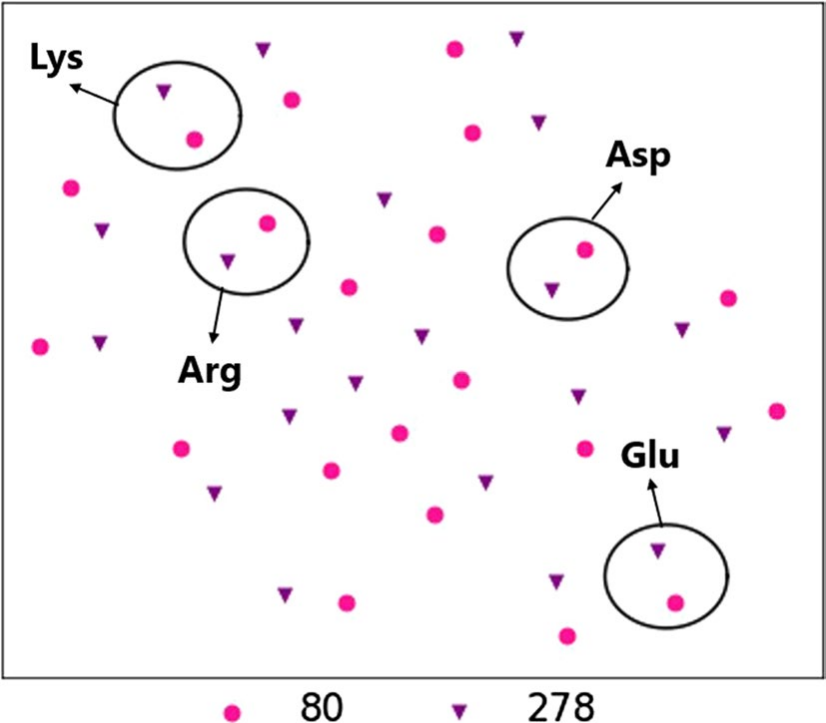
The $t-SNE$ coordinates of the embeddings. The sites 80 and 278 with different amino acids are labeled pink and purple, respectively

At last we compare the sites with different information gain, the top2 sites 278, 80 and the bottom2 sites 1 and 380. The combinative embeddings by sites and amino acids are colored differently in Fig. 8, where they are obviously clustered into two groups: sites with high information gain and sites with low information gain. We zoom in the details on site 80 and 278, and the results in Fig. 9 show that the pink and purple points co-exist in pairs. The points related to alkali-resistance amino acids are clustered together, while the points related to acid-resistance amino acids are clustered together. Comparatively, the points on alkali-resistance amino acids are closer than the acid-resistance amino acids. It illustrates that the embeddings have learned much information on distinguishing the amino acids and their influences on alkali-resistance functions.

**Fig. 10:**
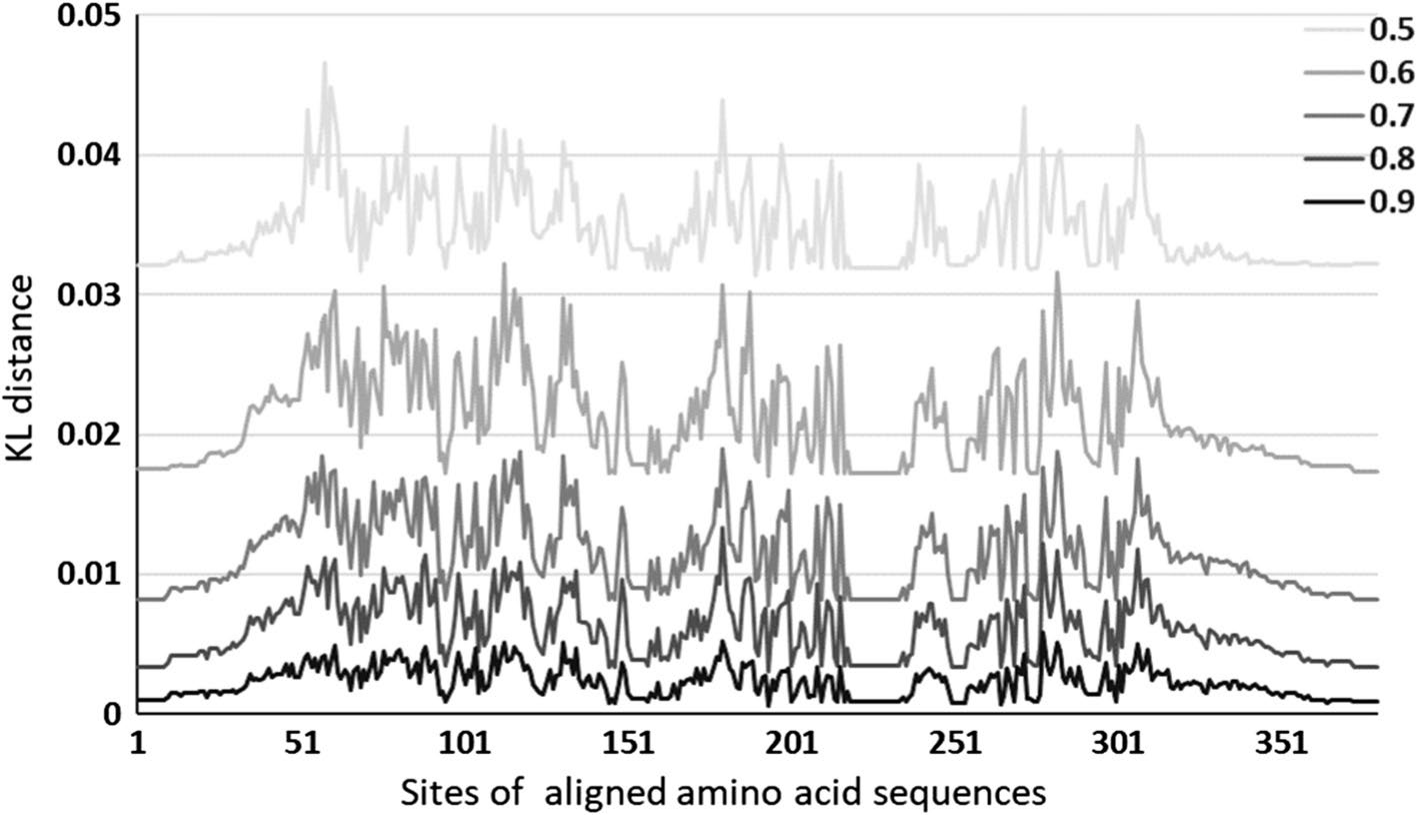
Evaluation on the approximate method

### Future work

By comparing the optimal pH between different amino acid sequences from the same family, we use the embeddings to predict the sequence with better alkali resistance. We also plan to design a more dedicated model by collecting and combining more datasets such that it can directly predict the optimal condition for a given sequence. Besides, we plan to analyze the optimal conditions of proteins in different families. Since they have different protein structures and aligned sequences, it would be more complex and challenging to analyze their conserved and non-conserved sequences and learning the embeddings of sites. Another interesting direction is to analyze multiple optimal conditions together. For different optimal conditions, the effectiveness of sites and amino acids in a protein may be different. The multi-objective analysis would help us find the relationships between different optimal conditions. This requires an elaborate design on the embeddings method so that they can contain more information to distinguish the features of different optimal conditions.

## Conclusion

In this paper, we propose an embedding method to represent the amino acids and the construct information as vectors in the latent space. These vectors contain information about the correlations between amino acids and sites in aligned amino acid sequences. Based on these embeddings, we then compare the two sequences, and determine the sequence with the more optimal conditions in the same family. Subsequently, we design a scoring method to suggest the site and direction of a mutation for an expected condition. We adopt the amino acid sequences in glycoside hydrolase GH11 family for the verification of our method. And we design two computational experiments and verify the results by previous wet experiments, which are to predict better performance in pH tolerance such as alkali resistance and to give a mutation suggestion. Compared with the traditional method, this method does not require the tertiary structure of a protein or the situation of free energy of the amino acid sequence, which is more efficient and effective.

These advantages attribute to two aspects. One is that we take into account both the correlations between the amino acid at a single site and the interaction between multiple-sites. Another is that the vectors we have learnt reveal the information about the optimal condition implied in amino acids sequence.

## Method

In this section, we present the details on the embedding method and the learning process.

### The embedding objectives

The optimal condition of an amino acid sequence is determined by which amino acids consist the enzyme and how they construct together in the sequence. Motivated by this point, we introduce two objective functions to learn the correlations between the optimal condition and the sequence: (1) the influence by one amino acid on each site. (2) the mutual influences by two amino acids on different sites. We propose an embedding method to combine these two objectives together by modeling the sites and the amino acids of a sequence into the vectors in the same latent space, denoted by ${\mathbf{v}} ,{\mathbf{c}} \in R^d$, respectively.

The first objective learns the correlations between the optimal condition and the type of amino acid on each site. We define the event (*k*, *a*) as amino acid $c_k^a\in A$ being on the $k{\rm th}, k\in [1..l]$ site of an aligned sequence $a \in {\mathbf {F}}$, and introduce the metric of *capability score* *s*(*k*, *a*) to evaluate how this event influences the optimal condition, formalized by:

2 $$\begin{aligned} s(k,a)={\mathbf{v}} _{\mathbf{k}} \cdot {\mathbf{c}} _{\mathbf{k}}^{\mathbf{a}} \end{aligned}$$

where ${\mathbf{v}} _k,{\mathbf{c}} _k^a\in R^d$ are the embeddings of site *k* and the amino acid $c_k^a$, respectively. A higher score means $c_k^a$ on site *k* resulting the sequence a better activity under a given condition.

Let **E** be the set of all occurrences of different events, namely the combination of amino acids and sites. We adopt the *softmax* function to model the probability of such an event.

3 $$\begin{aligned} p_\theta (k,a)=\frac{\exp (s(k,a ))}{\sum _{(k',a')\in {\mathbf{E}} } \exp (s(k',a'))} \end{aligned}$$

where $\theta$ denotes the parameters in this model. To maximize the likelihood of the occurrences, the objective loss function is formalized by

4 $$\begin{aligned} {\mathcal {L}}_T(\theta )=-\sum _{(k,a)\in {\mathbf{E}} }\log p_{\theta }(k,a) \end{aligned}$$

Since the normalization part in the denominator in Eq. 3 cost much computation, we use the Noise Contrastive Estimation (NCE) method, proposed by Gutmann et al. [19], to estimate the optimal parameters $\theta ^*$. It treats the normalization part as an additional parameter, denoted by *C*. The Eq. 3 is then re-written as:

5 $$\begin{aligned} p_\theta (k,a)=\exp (s(k,a)+C) \end{aligned}$$

According to NCE, we add artificially generated noise data to the training set. The parameters in probability density function and normalization part can be estimated by discriminating the original data and noise data. Let $p(D=1|(k,a),\theta )$ denote the probability that the optimal condition gets higher when $c_k^a\in A$ appears on site *k* in sequence $a \in {\mathbf {F}}$. Let $p(D=0|(k,a),\theta )$ denote the probability that the optimal condition gets lower when $c_k^a$ appears on site *k*.

6 $$\begin{aligned} p(D=1|(k,a),\theta )&= \frac{p_\theta (k,a)}{p_\theta (k,a)+p_n(k,a)}\nonumber \\&=\sigma (\log p_\theta (k,a)-\log p_n(k,a)) \end{aligned}$$

7 $$\begin{aligned} p(D=0|(k,a),\theta )&=1-p(D=1|(k,a),\theta )\nonumber \\&=1-\sigma (\log p_\theta (k,a)-\log p_n(k,a)) \end{aligned}$$

where $\sigma (x)=\frac{1}{1+\exp (-x)}$ is the *sigmoid* function and $p_n(k,a)$ is the artificial noise distribution.

We fit the model by maximizing the expectation of log-posterior probability over the mixture of observed samples and noise samples. The expectation and loss function are formulated by Eqs. 8 and 9:

8 $$\begin{aligned}{}&E_{p_\theta }[\log p(D=1|(k,a),\theta )]+E_{p_n}[\log p(D=0|(k,a),\theta )] \end{aligned}$$

9 $$\begin{aligned}{}&{\mathcal {L}}_T(\theta )=-[\log \sigma (\log p_\theta (k,a)-\log p_n(k,a))\nonumber \\&\quad +\log (1-\sigma (\log p_\theta (k',a')-\log p_n(k',a')))] \end{aligned}$$

The second embedding objective learns the correlations between the optimal condition and multiple amino acids appearing on different sites, namely how multiple elements together affect the optimal condition. Different from the single site analysis, the site *k* and the amino acid $c_k^a$ on it are considered together by concatenating their vectors,

10 $$\begin{aligned} {\mathbf{n}} _{\mathbf{k}}^{\mathbf{a}}={\mathbf{v}} _{\mathbf{k}}\oplus {\mathbf{c}} _{\mathbf{k}}^{\mathbf{a}} \end{aligned}$$

where ${\mathbf{v}} _k,{\mathbf{c}} _k^a\in R^d$ are the embeddings of site *k* and the amino acid $c_k^a$, respectively, and ${\mathbf{n}} _k^a \in R^{2d}$ is their joint embedding. We define the event $(n_i^a,n_j^a)$ as $c_i^a$ appears on site *i* and $c_j^a$ appears on site *j* in the sequence $a\in {\mathbf {F}}$. We also introduce the *suitability score* $s(n_i^a,n_j^a)$ to denote how much this event induces a better optimal condition.

11 $$\begin{aligned} s(n_i^a,n_j^a)={\mathbf{n}} _\mathbf{i }^{\mathbf{a}}\cdot {\mathbf{n}} _{\mathbf{j}}^{\mathbf{a}}=({\mathbf{v}} _\mathbf{i }\oplus {\mathbf{c}} _\mathbf{i }^{\mathbf{a}} )\cdot ({\mathbf{v}} _{\mathbf{j}}\oplus {\mathbf{c}} _{\mathbf{j}}^{\mathbf{a}}) \end{aligned}$$

A higher score means that the occurrence of event $(n_i^a,n_j^a)$ induces a better optimal condition and the probability is formalized by equation 12.

12 $$\begin{aligned} p_\theta (n_i^a,n_j^a)= \frac{\exp (s(n_i^a,n_j^a))}{ \sum _{(i',j')\in {\mathbf{E}} } \exp (s({n_{i'}^a},{n_{j'}^a}))} \end{aligned}$$

Similar to the discussion of Eq. 5–Eq.9, the loss function is formulated as follow:

13 $$\begin{aligned}{}&{\mathcal {L}}_C(\theta )=-[\log \sigma (\log p_\theta (n_i^a,n_j^a)-\log p_n(n_i^a,n_j^a))\nonumber \\&\quad +\log (1-\sigma (\log p_\theta ({n_i^a}',{n_j^a}')-\log p_n({n_i^a}',{n_j^a}')))] \end{aligned}$$

We model the loss function as a linear combination of the above two loss functions, where $\alpha \in [0,1]$ is a preference parameter.

14 $$\begin{aligned} {\mathcal {L}}(\theta )=\alpha \cdot {\mathcal {L}}_T(\theta )+(1-\alpha )\cdot {\mathcal {L}}_C(\theta ) \end{aligned}$$

### The embedding learning process

We adopt the stochastic gradient descent algorithm to optimize the parameters, proposed by *Adam* [20]. Given the set of amino acid sequences in a family, we partition them into two sets: one for training and another for test. For the expected optimal condition, the training set is further classified into the high optimal condition set $S_h$ and the low optimal condition set $S_l$. For example, if a higher pH is expected as the optimal condition, the amino acid sequences in $S_h$ have higher pH than those in $S_l$. We select a sequence *a* from $S_h$ and the amino acid $c_k^a$ on site *i* of *a* as a positive sample, i.e. the pair (*i*, *a*). A negative sample (*j*, *b*) is similarly chosen by site *j* on $b\in S_l$ so as to calculate the gradient of parameters. Taking Eq. 9 as an example, the gradient of the embedding ${\mathbf{v}} _i$ of site *i* in the loss function is calculated by:

15 $$\begin{aligned} \frac{\partial {\mathcal {L}}_{T}}{\partial {\mathbf{v}} _i}=-[\sigma (\log p_\theta (i,a)-\log p_n(i,a))-1]{\mathbf{c}} _i^a \end{aligned}$$

To calculate Eq.13, we need two positive samples (*i*, *a*), (*j*, *a*) to compose event $(n_i^a,n_j^a)$, and two negative samples $(i',b)$, $(j',b)$ to compose event $(n_{i'}^b,n_{j'}^b)$.

16 $$\begin{aligned} \frac{\partial {\mathcal {L}}_{C}}{\partial {\mathbf{v}} _{i}}=-[\sigma (\log p_\theta (n_i^a,n_j^a)-\log p_n(n_i^a,n_j^a))-1]{\mathbf{n}} _{\mathbf{j}}^{\mathbf{a}} \end{aligned}$$

In our experiments, we choose the higher alkali-resistance as the expected condition. Then the proteins with the optimal pH higher than 7 are classified into $S_h$ and others into $S_l$. When learning the embeddings, we select one or more samples from the two sets for each time. The learning process would stop until the objective function is converged.

### The approximation method

Since the size of a family dataset is small, we adopt the approximation method to generate more samples by the statistics on amino acids in the training dataset. To understand how this method works, we compare the probability distribution on the training samples and all samples by the KL distance, an often adopted metric to evaluate two probability distributions [21],

17 $$\begin{aligned} {KL-D}(P_i\parallel Q_i)=\sum _{c\in A}P_i(c)\cdot \log {\frac{P_i(c)}{Q_i(c)}} \end{aligned}$$

where $P_i$ and $Q_i$ denote the distribution on the amino acids at site *i* of an aligned sequence in two datasets, respectively. The smaller, the better. We set the proportion 50%, 60%, 70%, 80%, and 90%, respectively, to randomly select samples as the training part. The comparison results against the proportion are listed in Fig. 10, where X-axis represents the site and the Y-axis represents the KL distance. We can see that the difference decreases with an increasing size of training set. Together taking the embedding based optimal predication and mutant, as the results shown in the Experiment section, we have best results on 80% for learning.

### The quantitative score

After having the embeddings, we introduce the *quantitative score* as the predicted optimal condition for a specific sequence $a\in {\mathbf {F}}$. It considers two parts: the compatibility of the amino acid on each site and the influence induced by multiple amino acids on different sites. As the score functions *s*(*k*, *a*) and $s(n_i^a,n_j^a)$ defined in the Notions section, they are combined by the preference parameter $\alpha$, defined as:

18 $$\begin{aligned} Score(a)=\alpha \cdot \sum _{k=1}^ls(k,a_k)+(1-\alpha )\cdot \frac{\sum _{i,j=1}^ls(n_i^a,n_j^a)}{l} \end{aligned}$$

This score is used to predict the optimal condition of a new enzyme and to judge whether a mutant is appropriate for an expected condition.

## Data Availability

The data used in the experiments are from CAZy database, and the codes are available to provide. https://github.com/bj600800/EOCA.git

## Abbreviations

GH11: Glycoside hydrolase family 11
CAZy: Carbohydrate-active enzymes
NCE: Noise contrastive estimation
KL Distance: Kullback Leibler distance
t-SNE: Distributed stochastic neighbor embedding
SVR: Support vector regression
NN: Neural network

## Footnotes

1 A physics concept to describe the force that causes chemical reactions.

2 A sequence logo is created from a collection of aligned sequences and is widely adopted to depict the consensus sequence and diversity of the sequences, such as protein-binding sites in DNA or functional units in proteins. http://weblogo.threeplusone.com.

3 The CAZy database describes the families of structurally-related catalytic and carbohydrate-binding modules (or functional domains) of enzymes that degrade, modify, or create glycosidic bonds http://www.cazy.org/.
